# Arrest of labor secondary to large uterine fibroid

**DOI:** 10.1002/ccr3.8806

**Published:** 2024-04-20

**Authors:** Jesus Ruiz

**Affiliations:** ^1^ Department of Family Medicine University of North Carolina at Chapel Hill School of Medicine Chapel Hill North Carolina USA

**Keywords:** cesarean delivery, labor, myoma, pregnancy, uterine fibroids

## Abstract

**Key Clinical Message:**

Large uterine fibroids during pregnancy are associated with increased maternal and fetal complications. Large uterine fibroids should be kept in mind as a cause for arrest of labor and the need for cesarean delivery.

**Abstract:**

Uterine fibroids >5 cm in diameter are more likely to grow during pregnancy and cause obstetrical complications. We report a case of a large 9 cm subserosal uterine fibroid as the cause for the arrest of labor and the need for cesarean delivery.

## 1 CASE PRESENTATION

**FIGURE 1:**
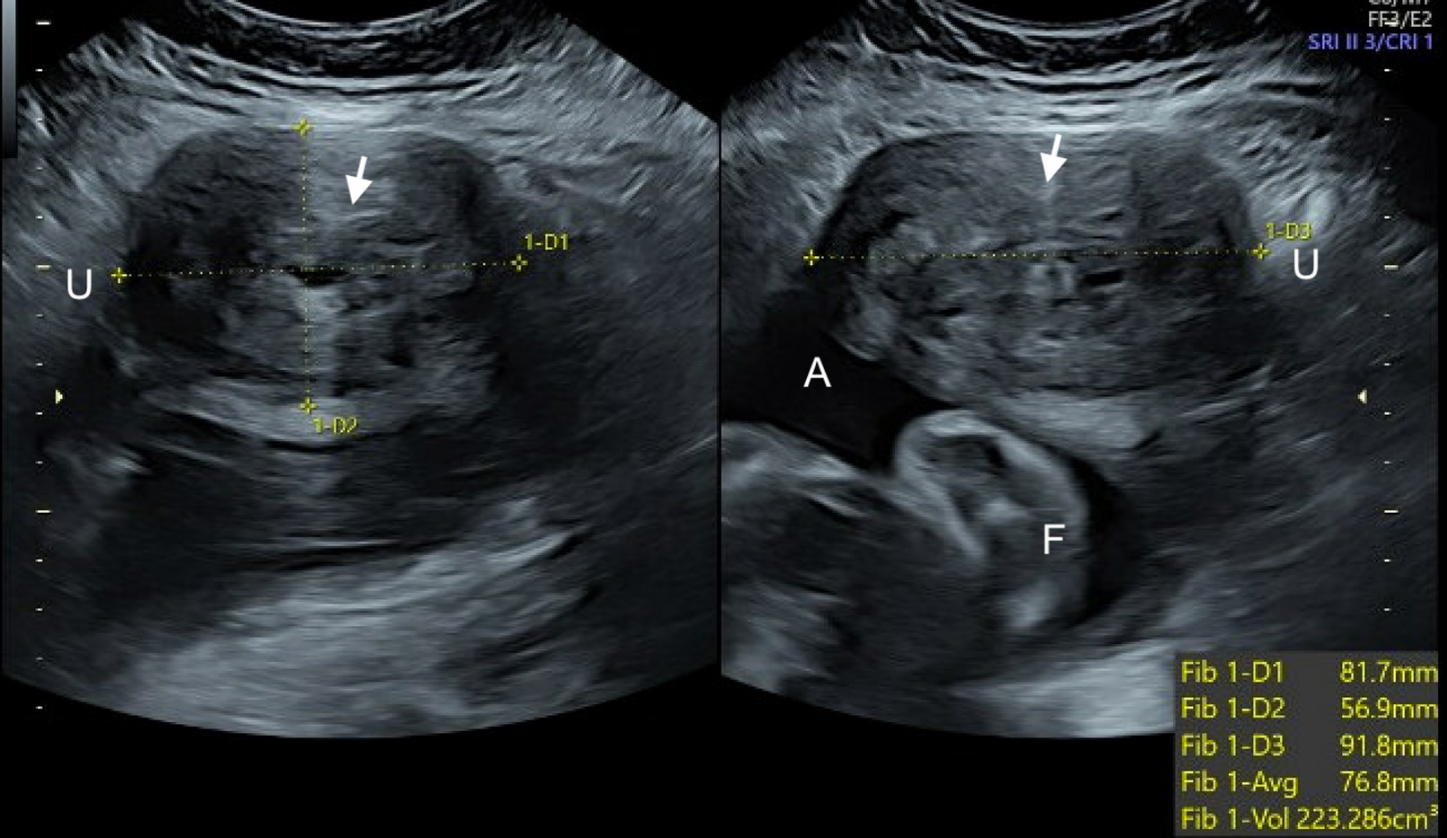
Prenatal ultrasound at 30 weeks of gestation showing a larger uterine fibroid (arrow), uterine wall (U), fetal parts (F), and amniotic fluid (A).

A 36‐year‐old female primigravida at 40 weeks and 2 days of gestation was admitted to labor and delivery for labor management. A 30‐week growth ultrasound showed normal fetal anatomy and growth as well as a large uterine fibroid on the anterior left lateral wall measuring 9.2 cm × 8.2 cm × 5.7 cm (Figure 1). The fibroid was not seen on prior ultrasounds at 15 and 22 weeks.

During labor, the patient progressed to 8 cm of cervical dilation. After 6 h of adequate uterine contractions, there was no further cervical dilation, with a repeat cervical exam revealing the left side of the cervix being swollen and edematous compared to the right side. A primary cesarean section was recommended secondary to arrest of labor and failure to dilate.

**FIGURE 2:**
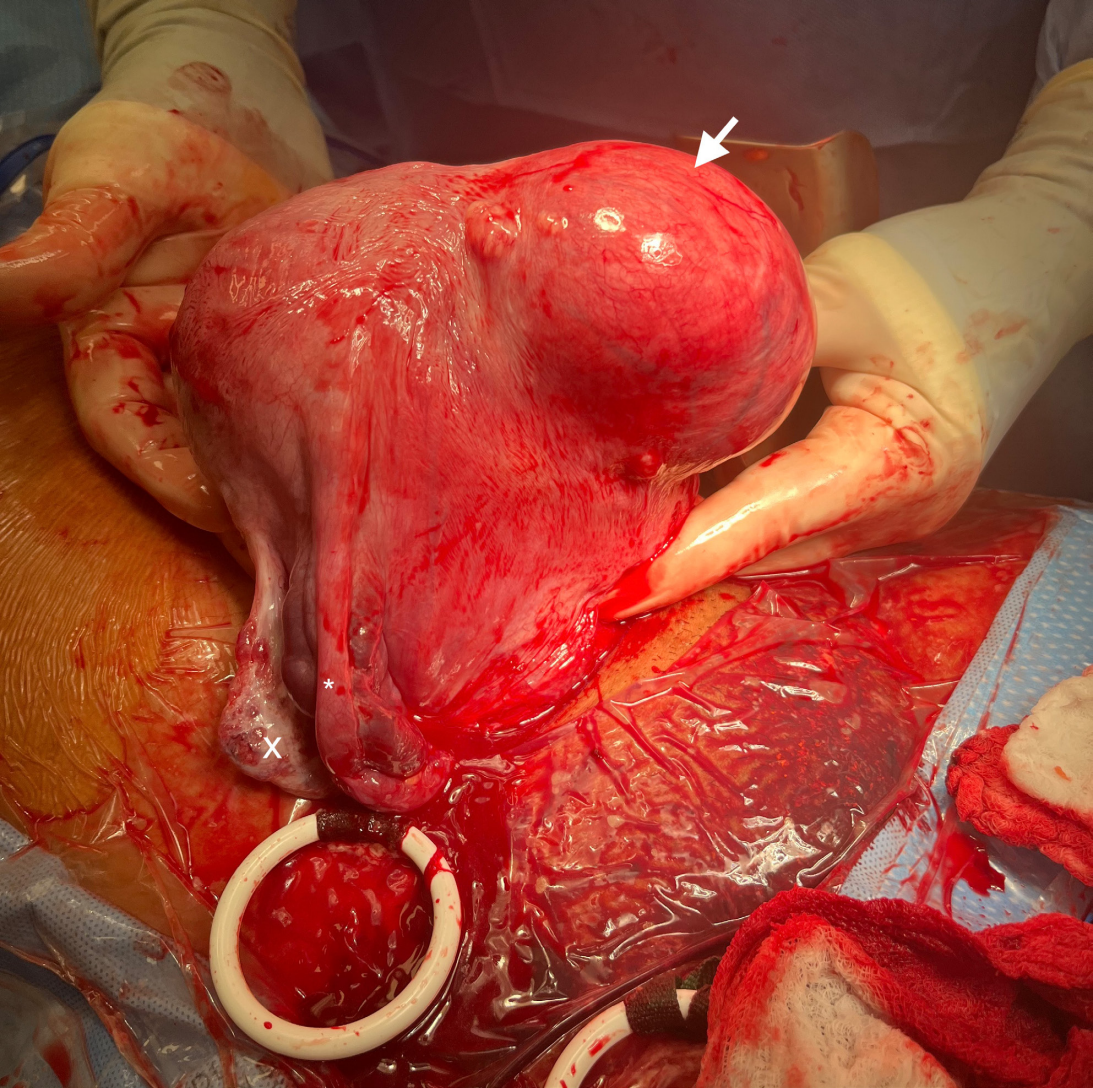
Uterus at the time of cesarean section with large 9 cm left anterior uterine subserosal fibroid (arrow) and normal right fallopian tube (*) and ovary (x).

After delivery of the newborn via cesarean section, the uterus was exteriorized and noted to have a large 9 cm × 8 cm × 5 cm mass on the left anterior lateral wall of the uterus (Figure 2). The rest of the uterus, ovaries, and fallopian tubes were unremarkable. The patient tolerated the procedure well without complications and was discharged from the hospital on postoperative day two in stable condition with the plan for a pelvic US at 6 weeks postpartum visit.

## 2 WHAT IS THE MOST LIKELY DIAGNOSIS?

The diagnosis of large subserosal uterine myoma was made at the time of cesarean section.

## 3 DISCUSSION

The diagnosis of uterine fibroids is made prenatally on physical exam or ultrasound; however, detection rates are limited. The prevalence of uterine fibroids during pregnancy is 1%–10% and is associated with a 10%–40% complication rate during pregnancy.^1^

Fibroids that are larger than 5 cm in diameter are more likely to grow during pregnancy and can lead to an increased risk of miscarriage, preterm labor, placenta abruption, malpresentation, labor dystocia, cesarean delivery, and postpartum hemorrhage.^1^, ^2^

Most women with uterine fibroids will deliver vaginally; however, uterine fibroids are a well‐known risk factor for cesarean delivery. Women with fibroids are 3.7 times more likely to need cesarean delivery due to fetal malpresentation and labor dystocia. Women with uterine fibroids during pregnancy are 2.5 times more likely to have fetal malpresentation and 2 times more likely to have labor dystocia. In most cases, uterine fibroids during pregnancy should not be considered a contraindication to trial of labor.^2^

Most authors agree that myomectomy at the time of cesarean section should be avoided, given the increased risk of severe hemorrhage and peripartum hysterectomy. New literature suggests that myomectomy during cesarean delivery could be considered in selected patients with careful consideration of several factors.^1^, ^2^, ^3^ We did not perform a myomectomy at the time of the cesarean section.

## AUTHOR CONTRIBUTIONS

**Jesus Ruiz:** Conceptualization; visualization; writing – original draft.

## FUNDING INFORMATION

No funding for the article.

## CONFLICT OF INTEREST STATEMENT

The author declares no conflict of interest.

## CONSENT

The patient gave written informed consent to publish this report in accordance with the journal's patient consent policy.

## PRESENTATIONS

None.

## Data Availability

Data sharing is not applicable to this article as no new data were created or analyzed in this study.
